# Physical Activity Is Associated with the Incidence of Depression in United States Adults from the NHANES 2013–18: A Cross-Sectional Study

**DOI:** 10.3390/healthcare12050552

**Published:** 2024-02-27

**Authors:** Damián Pereira-Payo, María Mendoza-Muñoz, Angel Denche-Zamorano, Ana Rubio-de la Osa, Miranda Moreno-Quintanilla, Raquel Pastor-Cisneros

**Affiliations:** 1 Health, Economy, Motricity and Education (HEME) Research Group, Faculty of Sport Sciences, University of Extremadura, 10003 Cáceres, Spain; dpereirapayo@unex.es (D.P.-P.); mmorenoq@alumnos.unex.es (M.M.-Q.); 2 Physical and Health Literacy and Health-Related Quality of Life (PHYQoL), Faculty of Sport Science, University of Extremadura, 10003 Cáceres, Spain; 3 Departamento de Desporto e Saúde, Escola de Saúde e Desenvolvimento Humano, Universidade de Évora, Largo dos Colegiais 2, 7000-645 Évora, Portugal; 4 Promoting a Healthy Society Research Group (PHeSO), Faculty of Sport Sciences, University of Extremadura, 10003 Cáceres, Spain; denchezamorano@unex.es (A.D.-Z.); raquelpc@unex.es (R.P.-C.); 5 Centro de Salud de Zafra, Servicio Extremeño de Salud, 06300 Zafra, Spain; arubiode@alumnos.unex.es

**Keywords:** mental health, social well-being, psychological disorders, sedentarism, physical fitness

## Abstract

The number of depression sufferers is rising globally. In the United States, 8% of adults over 20 years of age suffer from it, making it the most prevalent mental disorder in the country. Some lifestyle habits have been shown to favor or prevent the onset of depression; for instance, physical inactivity is associated with an increased likelihood of suffering depression, whilst multiple benefits have been attributed to performing physical activity (PA). This study aims to test whether there is a dependence between the prevalence of depression and PA, age, gender and educational level. The secondary objective was to identify the differentiating variables for depression and non-depression. This cross-sectional study is based on data from the NHANES 2013–2014, 2015–2016 and 2017–2018 editions. Some of the items in this survey were taken from preexisting questionnaires: the Patient Health Questionnaire-9 for depression screening and the Global Physical Activity Questionnaire (GPAQ) for the PA groups. The final sample was formed of 15,574 United States residents over 18 years old. After testing the data normality (*p* < 0.001), a descriptive analysis and the non-parametric chi-square test was conducted, as well as discriminant analysis. The results showed that there was an association between depression prevalence and PA (*p* < 0.001) in the general population and for both genders. Inactive participants had the highest prevalence of major depression and other depressive disorders. The discriminant analysis identified PA group (0.527), education level (0.761) and gender (−0.505) as significant variables that differentiate between participants with and without depression. The results of this research confirmed that a dependency relationship between PA group according to the GPAQ and depression prevalence according to the PHQ-9 existed in the United States adult population, and that PA group is a relevant variable to differentiate between depression sufferers and non-sufferers.

## 1. Introduction

Mental health, as defined by the World Health Organization (WHO), is the state of well-being of a person that allows them to develop their abilities, cope with their obligations, work productively and contribute to the community [1]. So, the absence of good mental health is considered to be one of the main causes of disability [2]. Given the increasing prevalence of mental health problems and the difficulties of therapeutic management, this problem constitutes a threat to global public health [3].

One of the consequences that can result from poor mental health is the development of mental disorders, of which depression has the highest proportion, with a total of more than 279 million people worldwide suffering from depression, a worrying increase from the figures of 170 million 30 years ago [2]. In the United States, as in the rest of the world, depression has also been catalogued as the most prevalent mental disorder, with more than 8% of the adults over 20 years of age suffering from it [4].

Depression is defined as a syndrome involving a state of “dysphoric mood” characterized by feeling sad, hopeless, irritable or losing interest and pleasure [5]. The symptoms of depression include a lack of interest and energy, a loss of concentration and feelings of guilt and worthlessness [6]. This illness can also have a detrimental influence on the daily work and social activities of the affected persons, with recurrent thoughts of death and even suicide being the most serious consequences [7]. The main risk factors for depression are younger age at onset, longer duration of depressive episodes and family history [8]. In addition, depression has been associated with a low level of social integration [8] as well as a low level of education [9]. Regarding sex, women have been reported to be more likely to suffer from depression than men [2,10]. From 2010 to 2018, the economic burden of adults with major depressive disorder increased by 37.9% in the United States [11]. In 2017, the total annual cost of medication-treated major depression disorder patients was 92.7 billion dollars in this country; these costs were derived from the healthcare burden (25.8 billion), unemployment burden (8.7 billion) and productivity burden (9.3 billion) [12]. Mental health, and specifically depression, entailed high costs for the United States’ society in direct costs, suicide-related costs and workplace costs [11]. Having healthy lifestyle habits has a direct repercussion on an individual’s physical and psychological health. For instance, practicing physical activity (PA) of any duration has been associated with improvements in lipid regulation, glycemic control, metabolic syndrome, multi-morbidity and all-cause mortality [13]. People from all ages benefit from having a physically active lifestyle, from children and adolescents [14] to adults and elderly people [15]. Even during pregnancy, its benefits are well documented [16]. The same occurs in pathologic populations, where cancer [17], diabetes [18], hypertension [19] and HIV [20] patients see benefits in their health from practicing regular PA.

Physical inactivity has been shown to increase the likelihood of depression [21,22,23]. Numerous studies have reported the beneficial role of PA in mental health [24,25,26], demonstrating its importance in improving depressive and anxious symptomatology [27], health-related quality of life [28], self-esteem, mood [29] and physical self-perception [30]. Bearing in mind the close relationship between depression and mood, it is worth noting that mood is strongly influenced by the secretion of endorphins [31]. Given the effect of regular PA increasing the expression of these hormones [32], this may be one of the key pathways of the antidepressant function of PA [33].

According to performed PA, people can be classified as inactives, for those who do not perform regular PA and have a sedentary lifestyle, and actives, for those who perform PA [34]. Depending on the volume, intensity and frequency of the performed PA, the active population can be divided into other subgroups [34]. Differences in health outcomes have been reported among different levels of PA [18,19]; those with less or no PA involvement have been found to have a greater prevalence of mental health problems [35,36,37] and non-communicable diseases [36,38]. Thus, given the proven positive effect of PA on mental health in general, and depression in particular, an association between performed PA and the prevalence of depression may exist.

Previous studies have investigated the possible association between depression and age group [39,40]. It has been shown that the incidence of depression remains present in all groups, and at the same time, increases with age. However, some specific risk factors, such as a low income level, have a stronger association at younger and less expected ages [41]. In addition to age group, female sex may be considered a risk factor for depression, as numerous previous research studies have reported a greater likelihood of depression in women than in their male counterparts [42,43,44].

Another factor that appears to increase the incidence of depression is educational level, as research has reported the association of lower educational attainment with a higher incidence of depression [45,46], mainly due to socio-economic disadvantage [47].

This study aims to test whether there is a dependence between the prevalence of depression and PA, age, gender and educational level. Additionally, the secondary objective of this research was to identify which variables differentiate between people who suffer from depression and those who do not. The study hypothesis is that declared physical activity is associated with a reduced prevalence of major depression and other depressive syndromes and that physical activity group, age, gender and education level are differentiating variables for classifying patients with or without depression.

## 2. Materials and Methods

### 2.1. Design

The present cross-sectional research is based on the National Health and Nutrition Examination Survey (NHANES) in its 2013–2014, 2015–2016 and 2017–2018 versions. This survey is conducted by the staff of the NHANES, which is formed of dietary and health interviewers, physicians and medical and health technicians. The NHANES is a program from the National Center for Health Statistics (NCHS) that aims to assess the health and nutritional status of adults and children in the United States, employing tools such as interviews and physical examinations [48]. This survey had been a continuous program since 1999, and in each edition, a nationally representative sample of around 5000 people is included. The NHANES data are publicly available for research purposes, as in the case of our study.

### 2.2. Participants

The NHANES 2013–14, 2015–16 and 2017–18 participant selection is based on a stratified, clustered, four-stage sampling system; all the relevant information about the sample design, calibration and weighting of data has been fully described [49]. The total number of participants in the NHANES 2013–18 added up to 29,400 people, with 14,452 males and 14,948 females. For this research, the following selection criteria were applied to the participants that made up the NHANES 2013–18 in order to select the final sample: being 18 years old or older and presenting valid data for the variables group of PA (items PAQ605, PAQ610, PAD615, PAQ620, PAQ625, PAD630, PAQ635, PAQ640, PAD645, PAQ650, PAQ655, PAD660, PAQ665, PAQ670, PAD675 and PAD680, which corresponded to the Global Physical Activity Questionnaire (GPAQ) [50]; depression (items DPQ010, DPQ020, DPQ030, DPQ040, DPQ050, DPQ060, DPQ070, DPQ080, DPQ090, which corresponded to the Patient Health Questionnaire-9 (PHQ-9) [51]; age (item RIDAGEYR); gender (item RIAGENDR) and education level (item DMDEDUC2 for adults over 20 years old and item DMDEDUC3 for adults 18 or 19 years old).

We excluded 13,826 individuals, of whom 4805 were excluded for not having valid data in the GPAQ and 9021 were excluded for not having valid answers in the PHQ-9. The final sample included in the current research consisted of 15,574 people (7571 males and 8003 females).

### 2.3. Variables

**Gender:** Male (coded as 1) or female (coded as 2).

**Age:** In years at the moment of interview.

**Age group:** This was derived from the variable “Age” and classifies participants into a specific age group depending on their age at the moment of interview. The groups formed are the following: 18–34 (for participants aged 18 to 34), 35–49 (for participants aged 35 to 49), 50–64 (for participants aged 50 to 64), 65–79 (for participants aged 65 to 79) and 80 and over (participants aged 80 or over in the moment of the interview).

**Education level:** For the participants aged 20 or above, this variable corresponded to the item “DMDEDUC2”, where the participants answered on what their highest grade or education level completed was; the answer options were: “Less than 9th grade”, “9th to 11th grade or 12th grade with no diploma”, “High school graduate/GED or equivalent”, “College or AA degree” and “College graduate or above”.

For the participants aged 18 or 19, this variable was derived from their answer to the item “DMDEDUC3”. The possible answers were 1st grade, 2nd grade, 3rd grade, 4th grade, 5th grade, “less than 5th grade”, 6th grade, 7th grade, 8th grade, “less than 9th grade” (all of the previously mentioned items corresponded to the group “Less than 9th grade”), 9th grade, 10th grade, 11th grade, “12th grade with no diploma” (these three options corresponded to the group “9th to 11th or 12th grade with no diploma”), “High school graduate”, “GED or equivalent” (these two options corresponded to the group “High school graduate/GED or equivalent”) and “More than high school” (which were classified as “College or AA degree”).

**PA group:** This variable was calculated using the GPAQ. Its 16 questions corresponded to the items PAQ605, PAQ610, PAD615, PAQ620, PAQ625, PAD630, PAQ635, PAQ640, PAD645, PAQ650, PAQ655, PAD660, PAQ665, PAQ670, PAD675 and PAD680 of the NHANES. Metabolic equivalents (METs) were calculated by attributing 8 METs to vigorous activities (PAQ605, PAQ610, PAD615, PAQ650, PAQ655 and PAD660), 4 METs to moderate activities (PAQ620, PAQ625, PAD630, PAQ665, PAQ670, and PAD675) and 3.3 METs to walking or cycling (PAQ635, PAQ640, PAD645) [52,53]. The GPAQ has been shown to be valid and reliable [50].

Participants were classified into a PA group in accordance with the following criteria [53]:

*High PA*: Participants that meet one of the following criteria: 1. Three or more days of vigorous physical activity or accumulating 1500 METs or 2. Seven or more days of any combination of light, moderate or vigorous physical activity that reaches 3000 METs [53].

*Moderate PA*: Participants that do not apply to the high PA group and meet one of the following criteria: 1. Three or more days of vigorous PA for at least 25 min per day; 2. Five or more days of moderate PA and/or walking for at least 30 min per day; 3. Five or more days of a combination of moderate and/or vigorous walking, reaching an expenditure of 600 METs [53].

*Low PA*: Participants that do not meet the criteria for the groups high PA and moderate PA but whose declared PA was higher than participants from the walkers and inactives groups.

*Walkers*: Participants that walked or cycled for 10 min continuously at least one day of the week but do not perform any other type of PA.

*Inactives*: Participants that did not perform any type of PA.

**Depression:** This was addressed using the Patient Health Questionnaire-9 (PHQ9) [54], a depression screening questionnaire that asks patients about the frequency with which they suffer depressive symptoms in a two-week period. For each of the nine items (DPQ010, DPQ020, DPQ030, DPQ040, DPQ050, DPQ060, DPQ070, DPQ080, DPQ090), patients answer on whether they have suffered a specific depressive symptom “not at all” (scoring 0 points), on “several days” (1 point), on “more than half the days” (2 points) or “nearly every day” (3 points) [55].

Participants were asked: “Over the last two weeks, how often have you been bothered by the following problems:”

“Little interest or pleasure in doing things?”

“Feeling down, depressed, or hopeless?”

“Trouble falling or staying asleep, or sleeping too much?”

“Feeling tired or having little energy?”

“Poor appetite or overeating?”

“Feeling bad about yourself—or that you are a failure or have let yourself or your family down?”

“Trouble concentrating on things, such as reading the newspaper or watching TV?”

“Moving or speaking so slowly that other people could have noticed? Or the opposite, being so fidgety or restless that you have been moving around a lot more than usual?”

“Thoughts that you would be better off dead or hurting yourself in some way?”

The PHQ-9 classifies patients into [55]:

*Major depression*: For patients who suffered at least 5 of the 9 depressive symptoms “more than half the days”, with one of these symptoms being depressed mood or anhedonia.

*Other depressive syndromes*: For patients that suffered 1 to 4 depressive symptoms “more than half the days”.

*No depression*: For patients who did not meet the criteria for a diagnosis of major depression or other depressive syndrome.

The PHQ-9’s validity and reliability has been proven [51,56,57].

**Any depression type:** This is a dichotomous variable derived from the variable depression; participants classified as having either major depression and other depression disorders according to the PHQ-9 were defined as “Yes” (to having any depression type), and participants designated as having no depression were defined as “No” (to not having any depression type).

### 2.4. Statistical Analysis

The statistical software SPSS in its 26th version (IBM SPSS, Chicago, IL, USA) was used to perform all of the statistical analysis. A 0.05 level of significance was assumed.

Firstly, the data distribution was addressed; the Kolmogorov–Smirnov test (*p* < 0.001) and histogram representation did not provide sufficient evidence to assume that the data followed a normal distribution, so no parametric test was used in the subsequent statistical procedures.

Ordinal variables such as age group, education level, PA group and depression were presented in their absolute and relative frequencies. The only continuous variable, which was age, was presented as a median and interquartile range, and as complementary data, the mean and standard deviation were also provided.

Secondly, the chi-square test was used to study the associations without interference between gender and the categorical variables and between major depression and other depressive syndromes and PA group. The post hoc pairwise z-test for independent proportions was employed to analyze the differences in the proportions between genders in the categorical variables and to analyze the differences in the proportions of major depression and other depressive syndromes among PA groups. To interpret the strength of the relationships (effect size) with major depression and other depressive syndromes, Cramer’s V was calculated.

The correlations between the PHQ-9 scores, having other depressive syndromes and having major depression and the variables age, gender, education level and PA group were studied using Spearman’s rho correlation coefficient.

Finally, a discriminant analysis was performed, using the variable “Any depression type” as the objective variable. The structure matrix coefficients allow us to identify variables that are significant to the discriminant function; all variables with a structure matrix coefficient higher than 0.30 or lower than −0.30 are considered to significantly contribute to the classification of cases and thus to the discriminant function. Canonical discriminant function coefficients are those that form the discriminant function, which classifies participants into “Yes” or “No” for depression type.

## 3. Results

The population under study had a median age of 48 years (IQR = 31), with no significant differences between men and women. No dependency relationships were found between age group and gender (*p* = 0.060), but significant differences in the distribution of the 35-to-49-year-old group were found, with more women than men in this age group (Table 1).

A relationship between education level and gender was found (*p* < 0.001), while significant differences in the distribution of education level by gender were found in all groups but “Less than 9th grade” and “College graduate or above”. PA group and gender were found to be associated (*p* < 0.001), with differences between males’ and females’ distribution in all PA groups but “Moderate PA”. Associations between the variables depression and gender and the variables “Any depression type” and gender were confirmed, with significant differences among distribution by gender in every subgroup of depression and “Any depression type”; females had a significantly greater prevalence in all of these groups (Table 1).

In Table 2, the relationship between depression prevalence and PA group was studied; the chi-square test showed a relationship between these two variables in the general population (<0.001), males (<0.001) and females (*p* < 0.001). Our results showed that participants from the PA groups that reported performing less PA, especially inactives, presented a significantly larger prevalence of other depressive syndromes and major depression than participants from PA groups with greater PA involvement. As a general tendency, the differences among the other PA groups (walkers, low PA, moderate PA and high PA) were not as pronounced as among inactives and these groups. These results suggested that greater PA may imply a reduced prevalence of major depression and other depressive syndromes.

Table 3 shows the correlations between the scores in the depression screener questionnaire PHQ-9, the prevalence of having other depressive syndromes and the prevalence of major depression and the variables gender, age, education level and PA group. Gender was significantly correlated with PHQ-9 scores (*p* < 0.001), other depressive syndromes (*p* < 0.001) and major depression (*p* < 0.001). Age was significantly correlated with having or not having other depressive syndromes (*p* < 0.001) and having major depression (*p* < 0.001) but did not correlate with the PHQ-9 scores (*p* = 0.132). On its end, education level was correlated with the PHQ-9 scores, having other depressive syndromes (*p* < 0.001) and suffering from major depression (*p* < 0.001). The same occurred for PA group, which was correlated with the PHQ-9 score (*p* < 0.001), other depressive syndromes (*p* < 0.001) and major depression (<0.001).

The discriminant analysis identified which variables were significant to differentiating between patients with major depression or other depressive symptoms and patients with no depression according to the PHQ-9. The present discriminant function successfully classified 60.2% of cases and pointed out the variables education level (0.761), PA group (0.527) and gender (−0.505) as significant variables for the classification of cases (Table 4). However, the variable age was not considered to significantly contribute to the classification of patients since its structure matrix coefficient was between −0.30 and 0.30.

Thus, among United States citizens, having a lower education level, being a woman or performing less PA indicated a greater likelihood of suffering from other depressive syndromes or major depression. Being older was also pointed out as an indicator of greater odds of suffering depression, but the variable age was not found significant in the discriminant analysis.

## 4. Discussion

The aim of the present research was to study the relationship between depression according to the PHQ-9 questionnaire and PA group according to the GPAQ in the adult population of the United States from the NHANES 2013–18. A discriminant analysis was performed in order to determine whether PA group was a significant variable for the classification of cases of patients with depression or with no depression. Depression and PA group were shown to be associated; this relationship without interference among variables was confirmed in the general population and in both genders. PA group was identified as a significant variable for the classification of cases of people with depression or without depression. Finally, the participants from the inactives group had the greatest major depression and other depressive syndrome prevalence in the general population, males and females.

PA group was found to have a significant association without interference with the prevalence of major depression and other depressive syndromes, and this phenomenon occurred in the general population (*p* < 0.001), males (*p* < 0.001) and females (*p* < 0.001). Among the PA groups, inactives, who did not perform any PA, were shown to have the highest prevalence of major depression and other depressive syndromes in the general population, males and females. The results are consistent with the study by Roshanaei-Moghaddam and colleagues [58], which also grouped participants by PA groups, finding a positive association between the more active groups and a lower prevalence of depression and depressive symptoms. Similarly, those who were inactive had a higher prevalence of depression [58]. As indicated by numerous studies, physical activity is a preventive factor for depression, as well as a good complementary treatment to reduce its symptoms [27,59,60,61,62].

Except in females, walkers did not present significant differences from inactives in the prevalence of major depression, but they did in the other depressive symptoms. This may be because although walking has been shown to have a significant effect on improving depressive symptoms in some populations, more research is still needed to establish the frequency, intensity, duration and type of effective walking interventions [63]. In older adults, walking has been shown to improve clinical depression [64], but the age of the participants may be an important factor in the effectiveness of walking interventions in terms of mental health benefit. Thus, older adults and the elderly population may experience greater benefits for mental health from physical activity in the form of walking than younger population groups.

In the general population and in females, the groups low PA, moderate PA and high PA had a significantly reduced prevalence of both types of depression than inactives, which suggests that performing greater PA is associated with a lower incidence of depression. These results are supported by several similar studies which found a relationship between active PA groups and a decreased prevalence of depression and depressive symptoms [59,60,65]. Various exercise interventions have suggested that greater improvements in psychological state can be achieved by increasing the amount of PA performed [56,57].

For males, there were no significant differences between inactives, walkers and the low PA group. But significant differences did exist between the moderate and high PA groups and inactives. This may suggest that men may need to perform more PA than women in order to experience the benefits of an active lifestyle on mental health. Some research supports the greater effect of more physically demanding PA recommendations in order to benefit mental health [22]. Regarding PA duration, an inverse association between PA duration and the incidence of moderate/severe depression symptoms has been found [22]. Additionally, among physically active men, replacing an hour of moderate PA with an hour of vigorous PA was seen to reduce the odds of depression by 32% [22].

The discriminant analysis developed a discriminant function that successfully classified 60.2% of participants into people with depression or without depression. Education level, PA group and gender were identified as significant variables for the classification of cases, which indicates that these variables differentiate between participants who suffer and do not suffer from this mental health problem. Thus, according to our results, having a lower education level, being female and performing less PA enhance the likelihood of suffering from major depression or other depressive syndromes according to the PHQ-9.

Performing PA has been shown to be associated with the incidence of depression. Participants with greater PA involvement are suggested to have a reduced prevalence of this mental health condition [66]. Different doses of exercise provoke different biological responses [27]; the duration, frequency and intensity of exercise have been shown to cause distinct neural responses, which could affect depressive symptoms differently [43,47]. Exercise interventions with more frequent and larger sessions have been shown to better stimulate the mechanisms through which exercise influences depressive symptoms [43,67]. Thus, research has suggested that greater volume, intensity and frequency can achieve greater improvements in psychological state [43,66]. The results of the present study confirm that physically inactive individuals are associated with greater incidences of depression, which is in line with the previous research [68] and supports the antidepressant effects attributed to physical activity [66]. Certainly, the optimal PA recommendations for mental health improvement are yet to be determined, but, most likely, a high component of individualization to suit the needs of each person and adhere them to an active lifestyle would be key.

Regarding education level, the research has reported the association of a lower education level with a greater incidence of depression. Pei et al. [45] indicated that people with a lower level of education have a higher prevalence of major depression and depressive symptoms [45]. The main reason for this may be that a lower education level tends to limit an individual’s access to higher-income positions, which could translate into certain socio-economic disadvantages that may end up affecting an individual’s psychological well-being [69,70]. Additionally, lower education and socio-economic levels have been reported to imply a lower level of participation in sports from childhood, which could deprive those in that situation from the psychological benefits of sports practice [71].

The greater likelihood of women suffering from depression has been confirmed over time in the United States population [72]. In fact, being a woman has been identified as a risk factor for depression worldwide [73,74,75]. New studies are being conducted to identify the sex differences in neurobiological processes that may cause these disparities in the prevalence of depression [76]. Male and female differences in conflict anxiety, fear processing, arousal, social avoidance, learned helplessness and anhedonia are some of the factors that have been studied and could explain sex differences in the prevalence of this mental health problem [76].

### 4.1. Practical Implications

Taking all of the above into account, increasing the amount of PA performed could mean a reduction in the risk of depression, especially in the most inactive PA groups. One of the possible practical implications of the present study could be launching awareness-raising messages in different US establishments, such as schools, universities and public places, about the importance of increasing PA, especially focusing on the improvement of mental health and the reduction in depression, with the purpose of reaching populations of all ages and socio-economic levels. In this way, initiatives related to active breaks could be promoted in public institutions, as well as in predominantly sedentary workplaces.

In addition, including sport scientists and PA professionals in health institutions, who can prescribe exercise adapted to the needs and/or pathologies of each person, would help to reduce the costs associated with the increase in mental disorders in the US population in the long term. Given that PA group is considered a significant variable for the classification of patients with or without depression, it should be defined as a protective factor in relation to mental health disorders. Therefore, exercise programs focused on sedentary populations should be developed, encouraging adherence to PA and exercise, seeking long-term physical and mental health improvements in adults with indications of depression. For people suffering from depression, it would be crucial to encourage them to practice PA through these programs so that they meet the PA recommendations of the World Health Organization [77] and the National Institute for Health and Clinical Excellence [78] for people with depression.

### 4.2. Limitations and Future Research Lines

One of the limitations of the present study is that depression is not diagnosed by a primary care provider, psychologist or psychiatrist; instead, participants are screened using the GHQ-9 questionnaire.

These are two potential opportunities for improvement that should be contemplated by future researchers to enhance the quality of their investigations: first, trying to implement objective procedures to directly measure the performed physical activity and also relying on medical professionals to diagnose depression.

On the other hand, it would be advisable to include objective PA data rather than self-reported information in subsequent studies. At the same time, it would be beneficial to include participants with previous depression as diagnosed by a specialist. Finally, another interesting future line of research would be to study the prevalence of depression and its association with performed physical activity in mental disorders other than depression.

## 5. Conclusions

The results of this research showed an association between PA group and the prevalence of major depression and other depressive syndromes. This association existed in the general population, males and females. Additionally, the discriminant analysis identified PA group, alongside education level and gender, as a significant variable to classify cases of patients with depression or without depression. The results showed that participants from the group “inactives” tended to have a greater prevalence of other depressive syndromes and major depression. Thus, the initial hypothesis was confirmed: a higher declared level of physical activity was found to be associated with a reduced prevalence of major depression and other depressive syndromes.

## Figures and Tables

**Table 1:** Characterization of the United States population aged 18 to 80 plus years from the NHANES 2013–18 and gender differences.

Variables				
**Age**	**Total (n = 15,574)**	**Male (n = 7571)**	**Female (n = 8003)**	
Median (IQR)	48.00 (31)	49.00 (32)	48.00 (30)	
Mean (SD)	48.39 (18.49)	48.47 (18.59)	48.31 (18.39)	
**Age groups (years)**	**Total (n = 15,574)**	**Male (n = 7571)**	**Female (n = 8003)**	***p***
18–34	4431 (28.5)	2191 (28.9)	2240 (28.0)	0.060
35–49	3571 (22.9)	1663 (22.0)	1908 (23.8) *	
50–64	3989 (25.6)	1947 (25.7)	2042 (25.5)	
65–79	2700 (17.3)	1346 (17.8)	1354 (16.9)	
80 and over	883 (5.7)	424 (5.6)	459 (5.7)	
**Education level**	**Total (n = 15,574)**	**Male (n = 7571)**	**Female (n = 8003)**	***p***
Less than 9th grade	1290 (8.3)	639 (8.4)	651 (8.1)	<0.001
9th to 11th or 12th grade with no diploma	2041 (13.1)	1074 (14.2)	967 (12.1) *	
High school graduate/GED or equivalent	3734 (24.0)	1901 (25.1)	1833 (22.9) *	
College or AA degree	4822 (31.0)	2153 (28.4)	2669 (33.4) *	
College graduate or above	3679 (23.6)	1801 (23.8)	1878 (23.5)	
**PA group**	**Total (n = 15,574)**	**Male (n = 7571)**	**Female (n = 8003)**	***p***
Inactives	3917 (25.2)	1521 (20.1)	2396 (29.9) *	<0.001
Walkers	962 (6.2)	419 (5.5)	543 (6.8) *	
Low PA	2811 (18.0)	1258 (16.6)	1553 (19.4) *	
Moderate PA	2660 (17.1)	1257 (16.6)	1403 (17.5)	
High PA	5224 (33.5)	3116 (41.2)	2108 (26.3) *	
**Depression**	**Total (n = 15,574)**	**Male (n = 7571)**	**Female (n = 8003)**	***p***
No depression	12,738 (81.8)	6435 (85.0)	6303 (78.8) *	<0.001
Other depressive syndromes	2186 (14.0)	893 (11.8)	1293 (16.2) *	
Major depression	650 (4.2)	243 (3.2)	407 (5.1) *	
**Any depression type**	**Total (n = 15,574)**	**Male (n = 7571)**	**Female (n = 8003)**	***p***
No	12,738 (81.8)	6435 (85.0)	6303 * (78.8)	<0.001
Yes	2836 (18.2)	1136 (15.0)	1700 * (21.2)	

*p* (*p*-value of chi-square test). Data (ordinal variables) are presented in absolute and relative frequencies (percentages represent relative frequencies of the PA groups (columns)). * (Significant differences between proportions of men and women at 95% z-test for independent proportions).

**Table 2:** Relationship between PA group and prevalence of depression in the United States population aged 18 to 80 plus years from the NHANES 2013–18, and gender differences.

PA Group										
General Population (n = 15,574)										
Depression Type	Inactives	Walkers	Low PA	Moderate PA	High PA	Total	*p*	Effect Size	X^2^	df
No depression	2994 (76.4) a	775 (80.6) b	2297 (81.7) b	2251 (84.6) c	4421 (84.6%) c	12,738 (81.8)	<0.001	0.068	142.27	8
Other depressive syndromes	672 (17.2) a	136 (14.1) b,c	402 (14.3) c	320 (12.0) b	656 (12.6) b	2186 (14.0)				
Major depression	251 (6.4) a	51 (5.3) a,b	112 (4.0) b,c	89 (3.3) c,d	147 (2.8) d	650 (4.2)				
**Males (n = 7571)**										
**Depression Type**	**Inactives**	**Walkers**	**Low PA**	**Moderate PA**	**High PA**	**Total**	***p***	**Effect Size**	**X^2^**	**df**
No depression	1229 (80.8) a	353 (84.2) a,b,c	1059 (84.2) c	1093 (87.0) b	2701 (86.7) b	6435 (85.0)	<0.001	0.058	51.70	8
Other depressive syndromes	220 (14.5) a	43 (10.3) b	152 (12.1) a,b	126 (10.0) b	352 (11.3) b	893 (11.8)				
Major sepression	72 (4.7) a	23 (5.5) a	47 (3.7) a,b	38 (3.0) b	63 (2.0) c	243 (3.2)				
**Females (n = 8003)**										
**Depression Type**	**Inactives**	**Walkers**	**Low PA**	**Moderate PA**	**High PA**	**Total**	***p***	**Effect Size**	**X^2^**	**df**
No depression	1765 (73.7) a	422 (77.7) a,b	1238 (79.7) b,c	1158 (82.5) c	1720 (81.6) c	6303 (78.8)	<0.001	0.067	72.79	8
Other depressive syndromes	452 (18.9) a	93 (17.1) a,b	250 (16.1) b	194 (13.8) b	304 (14.4) b	1293 (16.2)				
Major depression	179 (7.5) a	28 (5.2) a,b	65 (4.2) b	51 (3.6) b	84 (4.0) b	407 (5.1)				

Data (ordinal variables) are presented in absolute and relative frequencies (percentages represent relative frequencies of the PA groups (columns)). Effect size (effect size was calculated using Cramer’s V). a, b, c, d indicate significant differences between columns (if the same letter appears in two columns, there are no significant differences among them; if no letter appears in both columns, there are significant differences). Test: chi-square.

**Table 3:** Correlations among PHQ-9 scores, having other depressive syndromes and having major depression, and the variables gender, age, education level and PA group.

		PHQ-9 Scores	Other Depressive Syndromes (Yes/No)	Major Depression (Yes/No)
Gender	rho	0.139 *	−0.063 *	−0.047 *
	*p*-value	<0.001	<0.001	<0.001
Age	rho	−0.012	−0.028 *	−0.022 *
	*p*-value	0.132	0.001	0.005
Education level	rho	−0.097 *	0.093 *	0.081 *
	*p*-value	<0.001	<0.001	<0.001
PA group	rho	−0.078 *	0.051 *	0.069 *
	*p*-value	<0.001	<0.001	<0.001

rho: Spearman’s rho correlation coefficient. *p*-value: of Spearman’s rho. *: significant correlations among variables according to Spearman’s rho.

**Table 4:** Discriminant analysis to classify United States’ population aged 18 to 80 plus years from the NHANES 2013–18 into depression/no depression.

General Population (n = 15,566)			
Percentage of original cases correctly grouped			60.2%
**Structure Matrix Coefficients**			
**Variables**			**SC**
Educational level			0.761 **
PA group			0.527 **
Gender			−0.505 **
Age			−0.223
**Wilks’ Lambda**			
Wilks’ Lambda	Chi-square	Df	*p*
0.975	397.521	4	<0.001
**Eigenvalues**			
Eigenvalue	% of variance	Cumulative %	Canonical correlation
0.026	100.0	100.0	0.159
**Canonical Discriminant Function Coefficients**			
Age	X_1_		−0.006
Gender	X_2_		−0.972
Education level	X_3_		0.606
PA group	X_4_		0.210
(Constant)			−1.050
**Functions at Group Centroids (Mean Discriminant Function Scores)**			
No depression			0.076
Depression			−0.341
**Discriminant Function**			
Function = −1.050 − 0.006 ∗ X_1_ − 0.972 ∗ X_2_ + 0.606 ∗ X_3_ + 0.210 ∗ X_4_			

** Structure matrix coefficient is higher than 0.30 or lower than −0.30 (so that variable significantly contributed to the classification of cases); “Functions at Group Centroids” means discriminant function scores for each group; “Depression” = having “Major depressive syndrome” or “Other depressive syndromes” according to PHQ-9; “No depression” = not having any depressive syndrome according to PHQ-9.

## Data Availability

The used data were sourced from files that can be publicly accessed at https://www.cdc.gov/nchs/nhanes/index.htm (accessed on 16 July 2023).
